# Impacts of COVID-19 pandemic and public policies on corneal transplantations in Brazil

**DOI:** 10.5935/0004-2749.20230074

**Published:** 2022

**Authors:** Ana Maria Guimarães Garcia, Luciene Barbosa de Sousa, Alvio Isao Shiguematsu

**Affiliations:** 1 Associação Pan-Americana de Banco de Olhos, Rio de Janeiro, RJ, Brazil; 2 Department of Ophthalmology and Visual Sciences, Escola Paulista de Medicina, Universidade Federal de São Paulo, São Paulo, SP, Brazil; 3 Department of Ophthalmology and Eye Bank, Faculdade de Medicina de Botucatu, Universidade Estadual Paulista “Júlio de Mesquita Filho”, p>Botucatu, SP, Brazil

**Keywords:** Eye Banks, Cornea, Tissue donation, Corneal transplantation, COVID-19, Public policy, Brazil, Bancos de Olhos, Córnea, Doação de tecidos, Transplante de Córnea, COVID-19, Política pública, Brasil

## Abstract

**Purpose:**

The study aimed to evaluate the impact of coronavirus disease 2019 (COVID-19) pandemic and public policies on corneal donations and transplantations in Brazil and get reliable indicators to support effective measures for improving the system of obtaining, processing, distributing, using, and controlling donated ocular tissues.

**Methods:**

A questionnaire was applied by the Brazilian office of the Pan-American Association of Eye Banks (APABO) to Brazilian Eye Banks to collect data from January to August 2020 and generate reliable indicators about the impact of the COVID-19 pandemic on corneal donations and transplantations in Brazil.

**Results:**

Data from 37 Eye Banks showed that 76.1% of the 3,060 donations and 74.5% of the 3,167 transplants occurred in the pre-pandemic period. From the 6,052 processed corneas, 71.8% were provided for therapeutic purposes: 72.9% were transplanted, 26.1% ended up being discarded (45% of which qualified for optical transplantation), and 1% remained in stock in glycerin. Of the 1,706 corneas that could not be eligible for therapeutic use, 47.9% were excluded due to tissue conditions, 43.6% for serological reasons, 6.7% due to contraindications found in clinical history after retrieval, and 1.8% for other factors.

**Conclusions:**

The negative impact of the COVID-19 pandemic on corneal donations and transplantations in Brazil resulted from the recommendation of the Health Ministry to suspend the retrieval of ocular tissues from donors in cardiopulmonary arrest for almost six months. The indicators reveal the compelling requirement for updating both the classification and cornea provision criteria by the Eye Banks and improving the Brazilian corneal distribution system.

## Abstract (pt)

**Objetivos:**

Dimensionar o impacto da pandemia da COVID-19 nas doações e transplantes de córnea no Brasil e obter indicadores confiáveis para o embasamento de proposições de medidas efetivas para a manutenção e o aperfeiçoamento do sistema de obtenção, processamento, distribuição, utilização e controle dos tecidos oculares doados.

**Métodos:**

Um questionário foi enviado, pelo escritório Brasil da Associação Pan-Americana de Bancos de Olhos (APABO), aos Bancos de Olhos brasileiros. Dados de janeiro a agosto de 2020 foram coletados para gerar indicadores confiáveis sobre o impacto da pandemia da COVID-19 nas doações e transplantes de córnea no Brasil.

**Resultados:**

Dados de 37 Bancos de Olhos mostraram que 76,1% das 3.060 doações e 74,5% dos 3.167 transplantes aconteceram no período pré-pandemia. Das 6.052 córneas processadas 71,8% foram disponibilizadas para fins terapêuticos: 72,9% foram transplantadas, 26,1% acabaram sendo inviabilizadas (45% destas, classificadas para indicações ópticas) e 1%, em glicerina, permanecia em estoque. Das 1.706 córneas que não puderam ser disponibilizadas para uso terapêutico, 47,9% foram excluídas por fatores relacionados às condições dos tecidos, 43,6% por fatores sorológicos, 6,7% por contraindicações constatadas em histórico clínico após a captação e 1,8% por outros fatores.

**Conclusões:**

O impacto negativo da pandemia nas doações e transplantes de córnea no Brasil se deveu à recomendação do Ministério da Saúde de suspender, por quase seis meses, as captações de doadores em parada cardiorrespiratória. Os indicadores tornam evidente a necessidade de atualização dos critérios de classificação e disponibilização das córneas pelos Bancos de Olhos e do sistema nacional de distribuição destes tecidos.

## INTRODUCTION

From 2017 to 2019, Brazilian Eye Banks (EBs) obtained an annual average of 16,850 donors of ocular tissues (OTs), 31,791 processed corneas, and 17,205 corneal transplantations, according to the Tissue Banks Production Data Evaluation Reports, prepared by the Blood, Tissues, Cells and Organs Management (GSTCO), from *Agência Nacional de Vigilância Sanitária* (ANVISA)^(1)^. Data from the *Sistema Nacional de Transplantes* (SNT) showed that the average number of corneal transplantations in the same period was 15,380/year^(2)^.

A significant increase in corneal donations and transplantations was expected in 2020, as some EBs were expanding their teams and investing in educational campaigns. However, worldwide Severe Acute Respiratory Syndrome Coronavirus 2 (SARS-CoV-2) spread brought many uncertainties and concerns and, on 02/28/2020, the Brazilian office of the Pan-American Association of Eye Banks (APABO) released a guideline to the Brazilian EBs as a preventive measure recommending to include coronavirus disease 2019 (COVID-19) and the already known variants of coronavirus - Severe Acute Respiratory Syndrome (SARS) and Middle East Respiratory Syndrome (MERS) -, among the exclusion criteria for OT donors^(3)^. After World Health Organization (WHO) declared the COVID-19 pandemic on 03/11/2020^(4)^, the EBs in Brazil began restricting or even suspending activities as a security measure to the staff and the OT recipients until further scientific evidence. On 03/25/2020, the Health Ministry released Technical Note Nº. 25/2020^(5)^ recommending the suspension of OTs searching and removal from donors in cardiopulmonary arrest (CPA), pre-transplant outpatient appointments for people already enrolled on the waiting list, elective surgeries, while only brain death (BD) donors (with a negative reverse transcription polymerase chain reaction (RT-PCR) test), new case outpatient appointments, and emergency corneal transplants were allowed.

On 04/22/2020, the Health Ministry issued Technical Note Nº 34/2020^(6)^ reinforcing the recommendation that OT donations could only be obtained from BD donors without clinical or epidemiological COVID-19 features validated by a negative RT-PCR test for SARS-CoV-2 with a sample collected within 24 hours before OT removals. These recommendations were active for almost six months (until 09/18/2020) when Technical Note Nº 80/2020^(7)^ allowed the return of the elective surgeries (with specific protective measures) and the resumption of OT recoveries from donors in CPA. It also defined the RT-PCR test as optional.

Since the beginning of the pandemic, APABO has closely followed the massive drop in donations, the difficulties faced by the EBs, the negative impact on the recipients, and ophthalmological community concerns regarding prompt patient care and treatment.

On 8/7/2020, GSTCO/ANVISA released^(1)^ the first-semester partial data from 37 of the 51 EBs authorized by the Health Ministry, showing that 3,388 OT donors were obtained, and 3,171 corneal transplantations were performed, without specifying the monthly distribution of these numbers. For the same period, the SNT statistics^(8)^ indicated that 4,631 OT donors were obtained (30.4% in January, 31.8% in February, 23% in March, 7.1% in April, 4.1% in May, and 3.6% in June) and 3,930 corneal transplantations were performed (31.9% in January, 28.9% in February, 26.2% in March, 2.9% in April, 4.7% in May and 5.4% in June).

APABO prepared a questionnaire and requested, on 9/9/2020, EBs collaboration to provide monthly data from January to August 2020^(9)^ to accurately measure the impact of the Covid-19 pandemic on corneal donations and transplantations in Brazil and obtain reliable indicators to support propositions to the health authorities.

## METHODS

From the 51 EBs authorized to operate in Brazil, spread over 23 states and the Federal District, 50 were invited to participate, while APABO was unable to contact one of them.

The questionnaire was structured into nine topics (Chart 1), each containing a chart for the numerical insertion of monthly data from January to August 2020. The information requested on 9/9/2020 was organized to allow results standardization and unification and, consequently, common and accurate indicators generation. In three of the nine charts, gaps were available for indicating alternatives not included in the proposed justifications.

**Chart 1:** APABO Questionnaire to the Eye Banks

COVlD-19's PANDEMIC IMPACTS	
1. How many ocular tissue donors the Eye Bank obtained in the period (month by month)? ✓ Removals made by the Eye Bank x removals made by other teams ✓ Cardiopulmonary arrest donors x brain death donors	
2. How many corneas (whole globe and *in situ)* the Eye Bank obtained in the period (month by month)? ✓ Corneas removed by the Eye Bank team x corneas removed by other teams	
3. How many corneas classified for optical purposes were supplied to the State Transplant Center for distribution, how many were transplanted, and how many could not be transplanted (month by month)? ✓ Transplanted in home state x transplanted in another state	
4. From the corneas classified for optical purposes and supplied to the State Transplant Center for distribution, which could not be transplanted, the reasons for non-use were (month by month):	
✓ Tissue distribution delay by CNCDO ✓ Unavailable surgeons ✓ Unavailable patients ✓ Tissue transportation delay	✓ Temperature change during transportation ✓ Tissue classification change ✓ Others (describe below)
5. How many corneas classified for tectonic purposes (in Optisol-GS® or Eusol-C®) were supplied to the State Transplant Center for distribution, how many were transplanted, and how many could not be transplanted (month by month): ✓ Transplanted in home state x transplanted in another state	
6. From the corneas classified for tectonic purposes (in Optisol-GS® or Eusol-C®) and supplied to the State Transplant Center for distribution, which could not be transplanted, the reasons for non-use were (month by month):	
Tissue distribution delay by CNCDO ✓ Unavailable surgeons ✓ Unavailable patients ✓ Lack of patients for tectonic purposes ✓ Tissue transportation delay	Temperature change during transportation ✓ Tissue classification change ✓ Others (describe below)
7. How many corneas in glycerin were supplied to the State Transplant Center for distribution (consider those transferred from Optisol-GS® or Eusol-C® preservation media to glycerin and, also, those that were preserved directly in glycerin), month by month: ✓ Transplanted in home state x Transplanted in another state	
8. From the total corneas obtained (whole globe and In Situ), how many were not viable for therapeutic purposes and that could not be supplied to the State Transplant Center (month by month): ✓ Not preserved x preserved and not supplied	
9. From the total of non-viable corneas for therapeutic purposes, the reasons for not supplying the tissues to the State Transplant Center for distribution were (month by month):	
✓ Inappropriate tissue conditions ✓ Tissue processing failures ✓ Positive serologies ✓ Inconclusive serologies ✓ Inappropriate or insufficient blood sample ✓ Hemolysis ✓ Clinical history contraindications	✓ Tissue transportation delay to the Eye Bank ✓ Failures in tissue storage ✓ Inappropriate physical, clinic or social screening ✓ Others (describe below)

Source: Pan-American Association of Eye Banks (APABO)/Brazilian Office

(9)

When APABO requested EBs cooperation to collect the data, it committed to treating the information confidentially, compiling and presenting the overall results in a way to preserve each institution identity, avoiding comparisons or rankings, and disclosing the list of participating EB whenever the results are presented.

## RESULTS

From 50 EBs invited to participate, 44 answered: 40 sent the total data requested, two sent partial data, which could not be considered, one reported no activity during the assessed period, and one reported being inoperative. Of those who did not respond, three were inoperative, and APABO received no response from the other three. The data presented in this study are the responsibility of each unit and correspond to those provided by 80.5% of the institutions that recovered OTs in the first eight months of 2020, providing corneas for transplantation (37 EBs from 20 states and the Federal District). The data provided by one EB could not be used because, although complete, it presented discrepancies that the team was unable to rectify. Two EBs did not authorize data inclusion for scientific publication purposes, which did not affect obtained indicators interpretation because the quantity was reduced, but results evaluation remained unchanged.

The 37 EBs included in the study obtained 3,060 OT donors during the period (61.7% of the OT donors in Brazil, during the study time, according to the SNT^(8)^, which was a total of 6,052 processed corneas).

**Figure 1:**
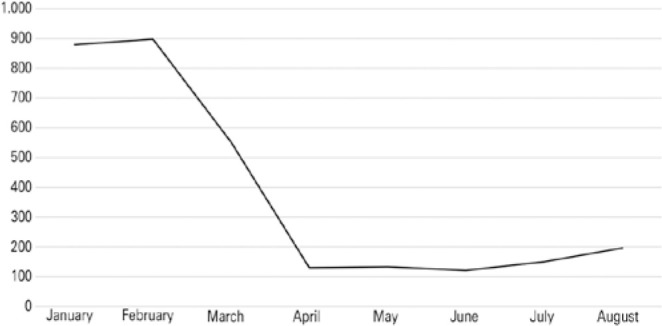
Ocular tissue donations obtained from January to August 2020.

Source: Pan-American Association of Eye Banks^(9)^

Figure 1 shows that 58.1% of the total donations in the period were recovered in January and February, 22.3% were recovered in March and April (18% in March and 4.3% in April), 8.3% were recovered in May and June, and 11.3% were recovered in July and August.

**Figure 2:**
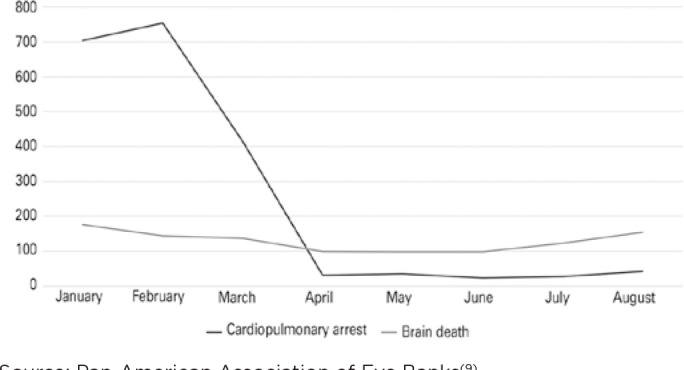
Ocular tissue donor conditions from January to August 2020.

Source: Pan-American Association of Eye Banks^(9)^

From the 3,060 donations, two-thirds (66.4%) were obtained from CPA donors, and the other third from BD donors. Retrieval from donors in CPA fell by 95.2% from the first to the second quarter, as shown in figure 2. In the first quarter, CPA donors represented 80.4% of donations. In the second quarter, CPA donors represented 23.2% of donations. Retrievals from BD donors also decreased from the first to the second quarter (a 35.5% drop): 22 EBs reported a reduction, 11 had a slight increase in BD donations (average increase of 5 donors per team), and four teams reported no change.

From 6,052 processed corneas, 4,346 (71.8%) were offered by the EBs to Organ Notification, Collection, and Distribution Centers (CNCDO) for patients on the waiting lists (73.5% for optical purposes and 26.5% for non-optical indications of which 4.6% were preserved in glycerin, extraordinarily, due to the restrictions imposed by the pandemic) and 1,706 (28.2%) could not be supplied by the EBs for therapeutic purposes.

From the total processed corneas, 3,167 (52.3%) were transplanted, what we called “General Utilization Index” (GUI): 84.7% of these for optical purposes and 15.3% for non-optical indications, of which 4.8% were in glycerin and were used in urgent cases.

From the 4,346 corneas supplied for therapeutic purposes, the same 3,167 that were transplanted correspond to 72.9% of what we called “Supplied Corneas Utilization Index (SUI)”.

**Figure 3:**
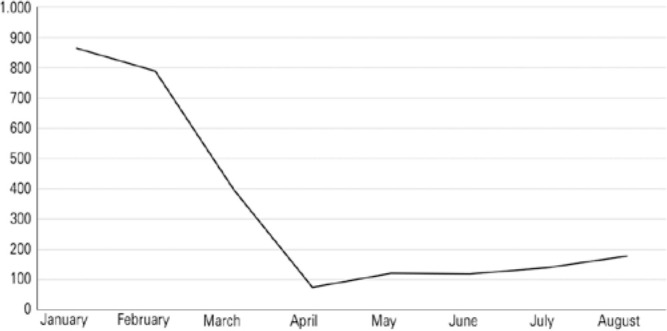
Transplants with corneas classified for optical indications from January to August 2020.

Source: Pan-American Association of Eye Banks^(9)^

From 3,194 corneas supplied for optical indications, 84% were transplanted: 76.4% were transplanted in the first quarter of 2020, as shown in figure 3.

**Figure 4:**
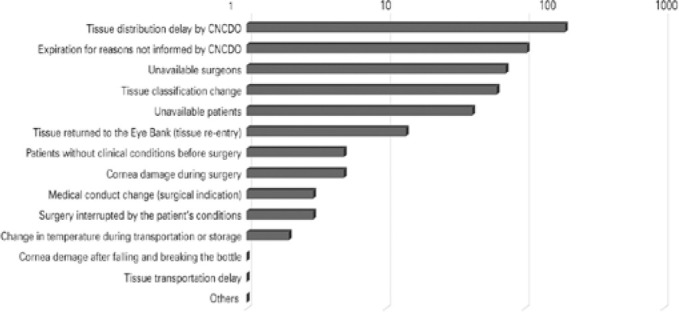
Reasons for not using corneas supplied to optical indications from January to August 2020.

Source: Pan-American Association of Eye Banks^(9)^

Figure 4 shows the reasons for not using 511 (16%) corneas supplied for optical transplantation (43.6% in the first bimester and 31% in the second).

CNCDO delay in optical corneas distribution was identified as the main reason for not using these tissues (38.4%), followed by corneal unfeasibility due to preservation period expiration (20.3%). In total, 58.7% of not used optical corneas became unviable due to problems faced by the CNCDO, as pointed out by 23 EBs from 12 states, and which might also have contributed to corneas unfeasibility that underwent classification changes during the preservation validity (12.3%). Unavailable surgeons represented 14.3% (71.2% in the first bimester), and unavailable patients represented 8.2% (52.4% in the first bimester).

A total of 1,152 corneas was supplied for non-optical indications: 954 (82.8%) in intermediate-term preservation medium (ITPM) and 198 in glycerin (a long-term preservation medium) on an extraordinary basis.

**Figure 5:**
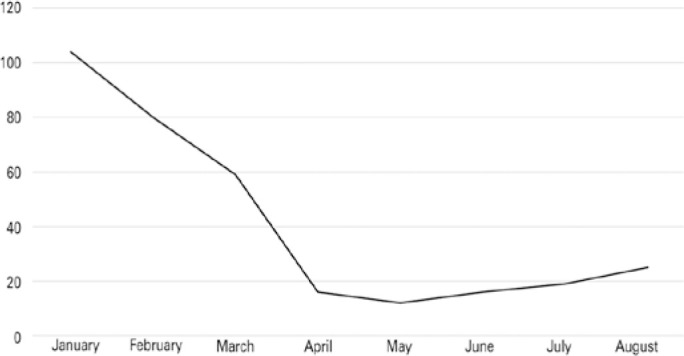
Transplants with corneas classified for non-optical (tectonic) indications supplied in ITPM from January to August 2020.

Source: Pan-American Association of Eye Banks^(9)^

From the 954 corneas in ITPM supplied for non-optical indications, 331 (34.7%) were transplanted, of which 73.5% were transplanted in the first quarter, as shown in figure 5.

**Figure 6:**
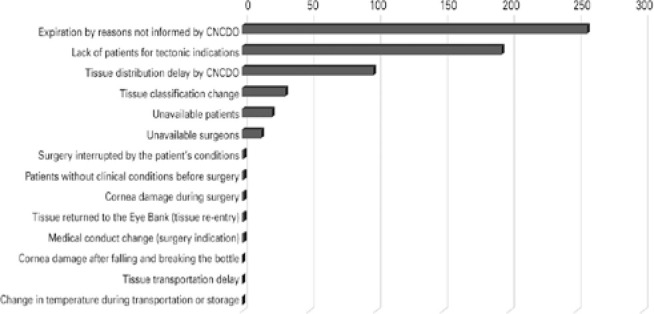
Reasons for not using corneas supplied in ITPM for tectonic indications from January to August 2020.

Source: Pan-American Association of Eye Banks^(9)^

From the 623 (65.3%) non-optical corneas in ITPM that were not transplanted, two-thirds (66.6%) became unviable in the first quarter. Figure 6 shows the main reasons for not using these corneas. In 41.4% of cases, their preservation period expired, and the EBs were not informed by the CNCDO about the reasons for their non-use. Lack of patients represented 31.1%, CNCDO delay in the distribution represented 15.7%, unavailable patients represented 3.5%, and unavailable surgeons represented 2.2%.

**Figure 7:**
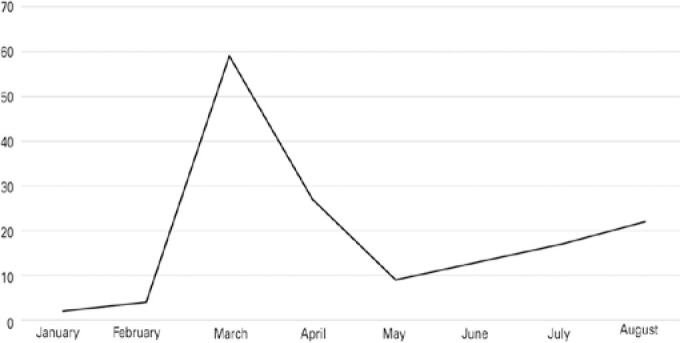
Transplants with corneas preserved in glycerin from January to August 2020.

Source: Pan-American Association of Eye Banks^(9)^

From the 198 corneas supplied in glycerin for emergency cases, 153 (77.3%) were transplanted (96.1% were transplanted after the pandemic was declared), and 45 (22.7%) remained in stock until the end of the study period, as shown in figure 7.

The EBs from the own states where the surgeries were performed provided 89.2% of the corneas for optical indications, 78.9% for non-optical indications with tissues in ITPM, and 80.4% for emergency surgeries with corneas in glycerin.

Of the 1,706 (28.2%) corneas that could not be supplied for therapeutic purposes, 55.5% were not preserved. The main reasons for this discard were: factors related to tissue conditions (47.9%), serological factors (43.6%), and contraindications in the clinical history after tissue removal (6.7%).

## DISCUSSION

Between 2017 and 2019, there were 1,825 fewer corneas on the average of corneal transplants published by the SNT, as compared to GSTCO/ANVISA data (from 94.1% of the 51 EBs authorized to operate). The conflicting data between the Health Ministry institutions is a consequence of the lack of a comprehensive, unified, and standardized information management system compatible with the peculiarities of the EBs. The relevant and standardized quantities precariousness hinders the generation of reliable indicators essential for qualitative control and guiding preventive and corrective measures.

With the start of the COVID-19 pandemic, the Health Ministry surprised the EBs community in Brazil with the adoption and maintenance of the recommendations that limited donations to BD cases, inserting the OTs in the context of organs and other donated tissues, for almost six months. The new standards did not consider corneal tissues particularities, scientific publications on SARS-Cov-2 and the cornea^(10-15)^, and international EB Associations recommendations, gathered in the Global Alliance of Eye Bank Associations (GAEBA)^(16)^, that specified strict screening criteria for OT retrievals^(3,17,18)^, without imposing restrictions on the conditions of the donors’ death.

With the restrictions, there was an 85.7% drop in OT donations from the first to the third bimester (considering the 37 EBs included in the study). According to the SNT indicators (from all EBs), the drop in OT donations was similar (87.6%).

Using the first bimester average in 2020 as a projection for the analyzed period (an average of donations, corneas processed, and transplants performed), we found that the rates achieved in the first eight months of 2020 were 57% lower compared to those that could have been reached without the interference from the COVID-19 pandemic. It is estimated that the 37 studied EBs were prevented from getting approximately 7,932 corneas, and thus, about 4,205 transplantations could not be performed. For the same period, if we consider SNT data, the same reduction rate (57%) regarding the projection is identified. The consequence is a 14.7% increase in the number of patients waiting for a corneal transplant, according to SNT data^(8)^ (12,205 patients on the waiting list on 01/31/2020, and 14,000 patients on 08/31/2020), which are numbers that are probably underreported due to the restrictions imposed by the pandemic (social distancing, limited appointments and care, and reduced donations and transplants, among others).

Both APABO and SNT data demonstrate that 74%80% of OT donations and transplantations occurred before the pandemic.

With donations suspension in CPA cases, higher activity in BD cases was expected. However, there was also a reduction (-35.5%) in BD donations from the first to the second quarter. Only 29.7% of the EBs showed an increase in BD donations after the pandemic had started.

The percentage of corneas available for therapeutic purposes (71.8%) is compatible and even higher than international standards, although this is a quantitative rather than qualitative indicator. If we consider EB indexes in the United States presented by Eye Bank Association of America (EBAA)^(19)^, we will find that the average of tissues supplied for therapeutic purposes from 2017 to 2019 was 66.9%, and GUI of that was 63.4%^(20)^_,_ while the index of Brazilian EBs, according to data from GSTCO/ANVISA^(1)^, was 54.1% (or 48.4%, if considering SNT data^(8)^) in the same period_._ From the data obtained in this survey, GUI was 52.3%. The lower corneas use in Brazil can be explained by SUI, which was 72.9% (while the average in the United States from 2017 to 2019 was 94.7%).

The 26.1% of corneas (optical and non-optical) supplied for therapeutic purposes and not used (disregarding 1% of the corneas provided in glycerin and that remained in stock) is high and corresponds to 1,134 not used viable corneas, whereas 76% of these were discarded before the pandemic. From this amount, 57.8% became unviable due to problems faced by the CNCDO, which may have also contributed to the unfeasibility of corneas that underwent alterations during the preservation validity period (8.4%); 17.1% were not used due to lack of patients waiting for non-optical corneas (for tectonic purposes); 7.7% were not used due to unavailable surgeons; 5.7% were not used due to unavailable patients; and 3.3% were not used due to unpredictable factors (for example, non-conformity in tissue storage, cornea damaged during surgical preparation, and patients’ clinical conditions).

Almost all these indexes (96.7%) are associated with the need to update classification and corneas availability criteria by the EB. The national tissue distribution system must be improved to enhance the matching between tissues supply with different surgical indications and enable potential recipients’ identification in any location in the country. Additionally, the criteria for registering patients on the waiting list and authorizing health establishments and specialized teams must be revised to ensure agility in tissues use. The distribution of corneas for optical purposes must not be interrupted on weekends and holidays by CNCDO to avoid tissue losses and preserve better corneas quality since the quality is inversely proportional to the preservation time. The loss of corneas for reasons that could be avoided has legal and ethical implications, not only because of the commitments with the donors’ families but also with the thousands of patients waiting for visual rehabilitation. Other aggravating factors are public resources waste and the compromise of humanitarian cause credibility.

Considering the number of not used corneas available in ITPM (1,134, of which 45% qualified for opti cal procedures), it is evident that the use of corneas preserved in glycerin could be avoided. For example, in the second bimester, in which 86 transplants were performed with glycerin-preserved corneas, 297 viable corneas preserved in ITPM were not used (158 classified as optical and 139 as non-optical). Even the limitations resulting from the pandemic regarding corneal distribution logistics, such as flights reduction or interruption, do not serve as a justification, as 64.9% of the EBs reported viable tissue loss between April and August (273 in total), a period in which 88 transplants were performed with corneas in glycerin (80.4% of surgeries with corneas obtained and processed by a local EB).

The results show that the waste of corneas qualified for therapeutic purposes occurs for reasons beyond the will and performance of the EB teams, considering unviable tissues and the tissues supplied to CNCDO. The non-preservation of 55.5% of the corneas that could not be supplied reflects the correct quality control and the coherence with the justifications for not supplying them for therapeutic purposes. The percentage of corneas that could not be offered but had been preserved (44.5%) is compatible with the factors that led to their non-use, which were mainly those related to serology.

After the COVID-19 pandemic has been declared, the highly negative impact on corneal donations and transplantations in Brazil resulted mainly from the recommendation of the Health Ministry for suspending OT retrievals from donors in CPA, the pre-transplant outpatient appointments for people already enrolled on the waiting list, and the elective surgeries for almost six months.

Before and during the pandemic, the results presented by the EB teams were consistent with international standards and reflected their serious work. The reasons that led to a high discard rate of corneas supplied by the EBs to the CNCDO for distribution are actually related not to the COVID-19 pandemic (76% occurred before the pandemic) but to problems faced by public managers to comply with the established policies.

The indicators reveal the compelling necessity to update both the classification and the provision criteria for corneas by the EBs and improve the Brazilian corneal distribution system.
